# Numerical and Experimental Study on the Performance of Thermoelectric Radiant Panel for Space Heating

**DOI:** 10.3390/ma13030550

**Published:** 2020-01-23

**Authors:** Hansol Lim, Jae-Weon Jeong

**Affiliations:** Department of Architectural Engineering, College of Engineering, Hanyang University, 222 Wangsimni-Ro, Seongdong-Gu, Seoul 04769, Korea; sollim0128@gmail.com

**Keywords:** Radiant heating, thermoelectric module, empirical modeling, finite difference method, non-vapor compression

## Abstract

The purpose of this study is to investigate the suitable operation and performance of a thermoelectric radiant panel (TERP) in the heating operation. First, the hypothesis was suggested that the heating operation of TERP can operate without a heat source at the cold side according to theoretical considerations. To prove this hypothesis, the thermal behavior of the TERP was investigated during the heating operation using a numerical simulation based on the finite difference method. The results indicated that it is possible to heat the radiant panel using a thermoelectric module without fan operation via the Joule effect. A mockup model of the TERP was constructed, and the numerical model and hypothesis were validated in experiment 1. Moreover, experiment 2 was performed to evaluate the necessity of fan operation in the heating operation of TERP regarding energy consumption. The results revealed that the TERP without fan operation showed the higher coefficient of performance (COP) in the heating season. After determining the suitable heating operation of the TERP, prediction models for the heating capacity and power consumption of the TERP were developed using the response surface methodology. Both models exhibited good R^2^ values of >0.94 and were validated within 10% error bounds in experimental cases. These prediction models are expected to be utilized in whole-building simulation programs for estimating the energy consumption of TERPs in the heating mode.

## 1. Introduction

Thermoelectric modules (TEMs) are attracting increasing attention as a non-vapor compression based technology [1,2,3,4,5] for preventing ozone depletion and global warming. The TEM applications in heating, ventilation and air conditioning (HVAC) can be categorized into air cooling/heating [6,7,8,9,10,11], liquid cooling/heating [12,13,14], and radiant cooling/heating [15,16,17,18,19,20]. Among the many applications of TEMs, the thermoelectric radiant panel (TERP) has much interest owing to its good constructability, compact size, and potential as a parallel cooling and heating device under stable thermal loads [21]. It has a limitation by the low performance value, which is evaluated as the figure of merit of thermoelectric materials (ZT) [22,23], however the recent advances in thermoelectric material make it possible to improve the ZT value [24,25,26,27]. Additionally, TERP does not require an additional heat exchanger for cooling or heating the liquid or air and it can be directly attached on the radiant cooling/heating surface. Therefore, there are less thermal losses between the surface of TEM and the radiant cooling/heating panel.

Lim and Jeong [15] proposed a dedicated outdoor air system with a water-cooled TERP, and the proposed system reduced the operation energy by 40.7% compared with the variable air volume system in an annual energy simulation. Luo et al. [16] proposed a building-integrated photovoltaic thermoelectric wall system for radiant cooling and heating, which exhibited annual energy savings of 29.19–62.94 kWh/m^2^ in Hong Kong in a simulation. Shen et al. [17] developed a mathematical model to optimize the TERP design for better cooling and heating performance. Their recommended number of TEMs was 16/m^2^ for TERP. Lim et al. [18] developed a design program for a TERP in a graphical user interface for easier accessibility. In their research, the triangular grid of the TEMs on the radiant panel exhibited a better temperature distribution for cooling compared with the normal rectangular grid of the TEMs. In addition, the empirical prediction models were developed to estimate the cooling performance of TERP on the purpose of design and simulation [19].

On the other hand, the heating operation of the TERP was still not established in the previous studies, even though the same TERP can be easily operated for heating purpose. The heating and cooling sides of the TEM can be simply switched by changing the direction of a direct current based on the Peltier effect [28]. Additionally, the TEM was identified as a good non-vapor compression HVAC technology for residential and commercial space heating in a previous report [29], because it has a higher coefficient of performance (COP) for heating than for cooling by reclaiming the heat from the cold side. Naturally, the heating COP of TEM is higher than that of the conventional electric heater as it is a heat pump.

In previous studies, TEMs have been employed for heating applications. The performance of a thermoelectric air heater for building was theoretically investigated using dynamic analysis [30]. The results indicated that a TEM can save the operation energy for heating by up to 64% compared with the electric heater. From the other thermoelectric heating system, it showed a COP of >2 when Δ*T* between the hot and cold sides was <20 °C [31]. Sun et al. [32] proposed a radiant heating terminal based on a TEM and a flat heat pipe and evaluated its performance through an experimental study. The experiments revealed that the COP of this radiant terminal ranged from 1.02 to 1.53 when Δ*T* between the cold and hot sides of the TEM was ranged from 12 to 38 °C. However, the previous studies were conducted under the low supply air and a low radiant surface temperature. Additionally, few radiant-heating approaches have been investigated using TEM. Therefore, it is necessary to investigate the characteristics of the TERP in the heating mode and evaluate its performance under various operation conditions.

In this study, the performance of the TERP for heating operation was analyzed via numerical and experimental investigations. First, the hypothesis was proposed that it is unnecessary to supply air (i.e., the heat source) to the cold side for the heating operation of TERP during the heating season, according to the theoretical background. Then, a thermal behavior of the TERP was numerically simulated using the finite difference method in the heating mode. In experiment 1, the numerical model and hypothesis were validated. Experiment 2 was conducted to estimate the necessity of fan operation in the heating mode of the TERP. Finally, empirical models were developed to predict the energy consumption and heating capacity of the TERP in the heating mode.

## 2. System Overview

**Figure 1:**
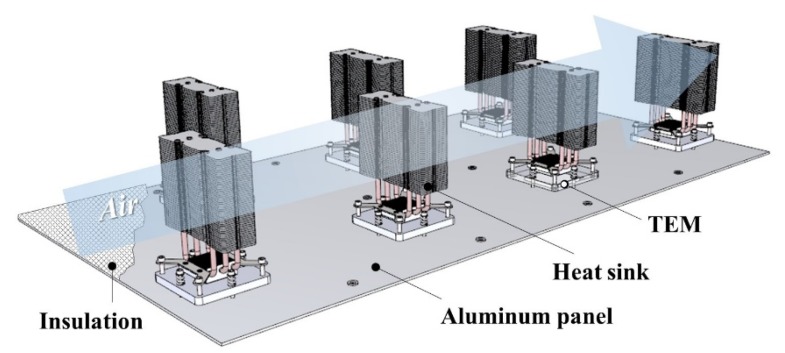
3D diagram of the thermoelectric radiant panel.

A TERP consists of an aluminum panel, insulation, heat sinks, and TEMs, as shown in Figure 1. The insulation is affixed on the rear side for reducing the heat transfer from the other side of the panel. The heat sinks are installed on the TEM to enhance the heat exchange area. In the cooling operation of TERP, TEM cooled the aluminum panel, and heat is rejected to the air side. Conversely, the aluminum panel is heated by the TEM in heating operation, and heat is absorbed from the air side. The cold and hot sides of TEM are simply switched by converting the direction of the electrode.

The amount of heat absorption and rejection at TEM are calculated using theoretical equations (Equations (1) and (2)) [28]. TEM works on the basis of the Peltier effect (*SIT*_c_ and *SIT*_h_), Joule effect (0.5*I*^2^*R*), and heat conduction (*K*Δ*T*). The driving force of the Peltier effect is an electromotive force, and heat conduction from the cold to the hot side of TEM. Simultaneously, heat is transferred from the hot side to the cold side of the TEM by the driving force of the temperature difference. Additionally, the TEM has its own electrical resistance; thus, heat is generated by the Joule effect.

(1) $$Q_{c,\;\mathrm{TEM}}=SIT_{c}-0.5I^{2}R-K\Delta T.$$

(2) $$Q_{h,\;\mathrm{TEM}}=SIT_{h}+0.5I^{2}R-K\Delta T\;.$$

The cooling operation of TERP employs outdoor air to remove the rejected heat for maintaining the cooling performance. In a previous study [15], a lower outdoor air temperature and higher air flow rate yielded a better COP, and it was essential to use a fan for cooling operation of TERP. If heat is not removed well at the hot side, the cooling performance decreases, because the amount of heat conduction exceeds the amount of heat flow via the Peltier effect.

**Figure 2:**
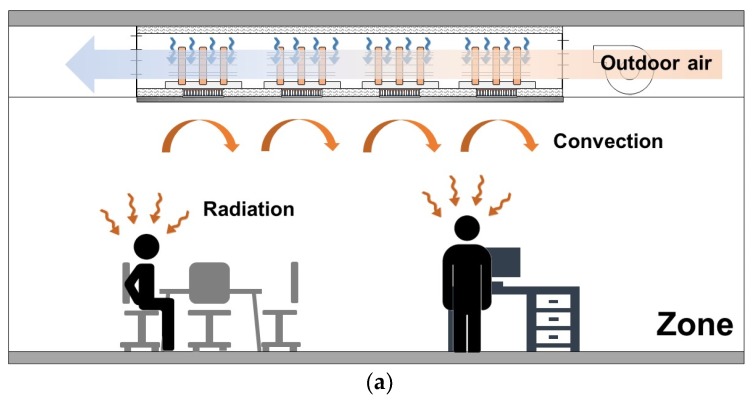

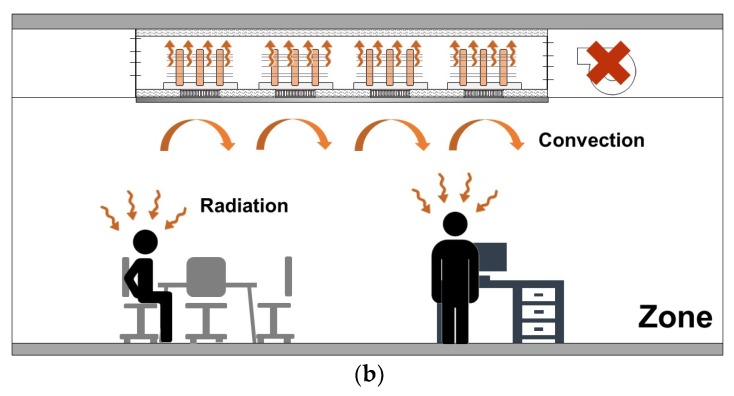
TERP in the heating mode: (**a**) heating mode with fan operation and (**b**) heating mode without fan operation.

In the heating mode, heat is absorbed from the outdoor air and transferred to the aluminum panel, similar with the air source heat pump (Figure 2a). The COP of the air source heat pump decreases with the temperature of the air source; moreover, the frosting problem may occur when the outdoor air temperature is lower than the freezing point [33,34]. For solving this, the vapor-compression cycle-based heat pump uses pre-dehumidification of the inlet air, ultrasonic vibration, and surface treatment of the heat exchanger [35,36,37]. Therefore, it can be assumed that the low temperature air source at the cold side may hinder the heating performance of TERP. If the hypothesis is correct, the fan operation is unnecessary (Figure 2b).

Between the surface of heating panel and air in the room, the heat is transferred through radiation and convection (Figure 2). However, the natural convection can be very weak if there is no air movement because the air is stagnant due to the stratification. Therefore, it is better making air movement to induce a forced convection.

## 3. Numerical Model

A numerical model for the TERP was established to investigate the thermal behavior of the TERP in the heating mode without fan operation (i.e., without an air source). The principle of the heating mode without fan operation in the TERP can be confirmed by validating the results of the numerical model and in situ experiments.

### 3.1. Simulation Overview

A two-dimensional (2D) numerical simulation was conducted using the finite difference method. The main purpose of numerical simulation was to confirm the working process of TERP for the vertical heat transfer through TEM, therefore the 2D analysis was suitable for this study. The thermal behavior of the base plate, insulation, aluminum panel, and TEM was analyzed. The air temperature in the duct was assumed to be constant, i.e., equal to the ambient temperature, which was assumed to be 26 °C. In the initial condition, all the nodes were set to the ambient temperature. For the boundary contacts with insulation, the Neumann boundary condition (BC) was used. The symmetric BC was applied at the center of the TEM to reduce the calculation time. Additionally, for the boundary contacts with the air side, a constant BC was used, according to the assumptions that the air temperatures of the room air and duct were constant. Binary control in an open-loop system was used to achieve the target surface temperature of TERP (i.e., on/off control). In each time step of the simulation, the mean surface temperature was calculated to investigate the operation of the TEM. Power was supplied, unless the mean surface temperature was higher than the target value with a 1 °C deadband.

### 3.2. TEM Model

The thermophysical properties of the TEM were simulated according to the properties of the TEM in Table 1 using the semi-black box model [38]. The thermal conductivity (*K*), electrical resistivity (*R*), and Seebeck coefficient (*S*) of the TEM are given by Equations (3)–(5), respectively. When the input current is determined, the cooling and heating capacities can be derived using Equations (1) and (2) with the calculated thermophysical properties of the TEM. Additionally, the input voltage and power consumption can be derived using Equations (6) and (7).

(3) $$K=\frac{{\left(T_{h}-\Delta T_{\max}\right)}^{2}}{T_{h}^{2}}\frac{Q_{\max}}{\Delta T_{\max}}.$$

(4) $$R=\frac{2{\left(T_{h}-\Delta T_{\max}\right)}^{2}}{T_{h}^{2}}\frac{Q_{\max}}{I_{\max}^{2}}.$$

(5) $$S=\frac{2Q_{\max}\left(T_{h}-\Delta T_{\max}\right)}{T_{h}^{2}I_{\max}}.$$

(6) V=IR+SΔT.

(7) $$P=I^{2}R+SI\Delta T=Q_{h,\mathrm{TEM}}-Q_{c,\mathrm{TEM}}.$$

### 3.3. Heat-Transfer Model

**Figure 3:**
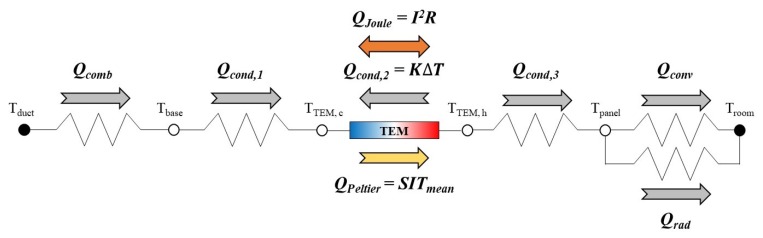
Steady-state heat flows through the TEM in the heating mode of the TERP.

In Figure 3, the steady-state heat flows of the TERP from the duct air to the room air through the TEM are illustrated. There are three heat flows in the TEM as described in Section 2. The heat is transferred by heat convection and radiation from the surface of the panel to the room. The heat is conducted from the surfaces of the TEM to the aluminum panel and the base plate of the heat sink, respectively. At the heat fins, the heat is transferred by convection from the air in a duct to the fins, and heat is conducted to the base plate.

The heat convection and radiation at the surface of the TERP can be determined using Equations (8)–(16). The Reynolds (*Re*) and Nusselt (*Nu*) number of the room air at the panel surface were calculated using Equations (8) and (9), respectively [39,40,41]. The characteristic length (*L_c_*) was 0.4 m, which is equal to the length of the panel (*L_panel_*) because the room air on the surface of the panel is an external fluid [42]. The Grashof number (*Gr*) was derived using Equation (10), and if $\frac{Gr}{Re^{2}}$ < 1.1, the heat-convection coefficient (*h_conv_*) could be directly determined using Equation (13). Unless it is <1.1, the convection should be treated as natural convection, and the correction equations for the Nusselt number (*Nu*) should be used in Equations (11) and (12) [43,44]. Consequently, the amount of heat convection can be determined using Equation (14). These equations were also used to calculate the convection coefficient at the surface of the insulation and the base plate without heat fins in the simulation.

(8) $$Re=\;\frac{\rho_{a}v_{a}L_{c}}{\mu_{a}}.$$

(9) $$Nu=\begin{array}{c} 0.664Re^{\frac{1}{2}}Pr^{\frac{1}{3}}\;(Re<5\times 10^{5})\\ 0.037Re^{\frac{4}{5}}Pr^{\frac{1}{3}}\;\left(Re\geq 5\times 10^{5}\right) \end{array}.$$

(10) $$Gr=\frac{g\beta \left(T_{\mathrm{panel}}-T_{\mathrm{room}}\right)L_{c}{}^{3}}{\nu_{a}{}^{2}}.$$

(11) Ra=Gr×Pr.

(12) $$Nu=\begin{array}{c} 0.54Ra^{\frac{1}{4}}\;(Ra<10^{7})\\ 0.15Ra^{\frac{1}{3}}\;\left(Ra\geq 10^{7}\right) \end{array}.$$

(13) $$h_{\mathrm{conv}}=\frac{\kappa_{a}Nu}{L_{c}}.$$

(14) $$Q_{\mathrm{conv}}=h_{\mathrm{conv}}A_{\mathrm{panel}}\left(T_{\mathrm{panel}}-T_{\mathrm{room}}\right).$$

The heat-radiation coefficient was determined using Equation (15) [42]. The mean radiant temperature (*T*_MRT_) was considered being equal to the room air temperature (*T*_room_) [45]. This assumption is practicable in the design stage because the radiant panel is usually installed for the internal zone, which has low radiation asymmetry [21]. The surface of the radiant panel was finished with a white paper sheet whose emissivity (*ε*_panel_) was 0.95. Finally, the amount of heat radiation from the panel to the room can be calculated using Equation (16).

(15) $$h_{\mathrm{rad}}=\varepsilon_{\mathrm{panel}}\sigma \left(T_{\mathrm{MRT}}{}^{2}+T_{\mathrm{panel}}{}^{2}\right)\left(T_{\mathrm{MRT}}+T_{\mathrm{panel}}\right).$$

(16) $$Q_{\mathrm{rad}}=h_{\mathrm{rad}}A_{\mathrm{panel}}\left(T_{\mathrm{panel}}-T_{\mathrm{MRT}}\right).$$

The amount of heat conduction at the hot side and cold side of TEM to the panel and base plate (*Q*_cond,1_ and *Q*_cond,3_) was calculated according to the simple heat conduction equations of Equations (17) and (18), independently. Additionally, the rejected and absorbed heat of TEM were derived using Equations (1) and (2), respectively.

(17) $$Q_{\mathrm{cond},\;1}=\kappa_{\mathrm{Al}}\frac{A_{\mathrm{panel}}}{\tau_{\mathrm{base}}}\left(T_{\mathrm{base}}-T_{\mathrm{TEM},c}\right).$$

(18) $$Q_{\mathrm{cond},3}=\kappa_{\mathrm{Al}}\frac{A_{\mathrm{panel}}}{\tau_{\mathrm{panel}}}\left(T_{\mathrm{TEM},\;h}-T_{\mathrm{panel}}\right)\;.$$

### 3.4. Discretization Using the Finite Difference Method

The governing equations were established (Equations (19)–(23)) using the finite difference method. Equation (19) is a governing equation for non-boundary nodes. The properties were applied according to the location of the node, such as the density (ρ), specific heat capacity (*C*_p_), and heat conductivity (*κ*). Equations (20)–(23) are the governing equations for the top surface of the base plate, cold side of the TEM, hot side of the TEM, and bottom surface of the aluminum panel, respectively.

(19) $$\rho C_{p}\frac{\partial T}{\partial t}=\kappa \left(\frac{\partial^{2}T}{\partial x^{2}}+\frac{\partial^{2}T}{\partial y^{2}}\right).$$

(20) $$\rho_{\mathrm{Al}}C_{p,\mathrm{Al}}\frac{\partial T_{\mathrm{base}}}{\partial t}=\kappa_{\mathrm{Al}}\left(\frac{\partial^{2}T_{\mathrm{base}}}{\partial x^{2}}+\frac{\partial^{2}T_{\mathrm{base}}}{\partial y^{2}}\right)+\frac{Q_{\mathrm{comb}}}{\partial x\partial yW_{\mathrm{panel}}}.$$

(21) $$\rho_{\mathrm{TEM}}C_{p,\mathrm{TEM}}\frac{\partial T_{\mathrm{TEM}}}{\partial t}=K\left(\frac{\partial^{2}T_{\mathrm{TEM}}}{\partial x^{2}}+\frac{\partial^{2}T_{\mathrm{TEM}}}{\partial y^{2}}\right)+\frac{Q_{c,\;\mathrm{TEM}}}{\partial x\partial yW_{\mathrm{panel}}}.$$

(22) $$\rho_{\mathrm{TEM}}C_{p,\mathrm{TEM}}\frac{\partial T_{\mathrm{TEM}}}{\partial t}=K\left(\frac{\partial^{2}T_{\mathrm{TEM}}}{\partial x^{2}}+\frac{\partial^{2}T_{\mathrm{TEM}}}{\partial y^{2}}\right)+\frac{Q_{h,\;\mathrm{TEM}}}{\partial x\partial yW_{\mathrm{panel}}}.$$

(23) $$\rho_{\mathrm{Al}}C_{p,\mathrm{Al}}\frac{\partial T_{\mathrm{panel}}}{\partial t}=\kappa_{\mathrm{Al}}\left(\frac{\partial^{2}T_{\mathrm{panel}}}{\partial x^{2}}+\frac{\partial^{2}T_{\mathrm{panel}}}{\partial y^{2}}\right)+\frac{\left(Q_{\mathrm{conv}}+Q_{\mathrm{rad}}\right)}{\partial x\partial yW_{\mathrm{panel}}}.$$

**Figure 4:**
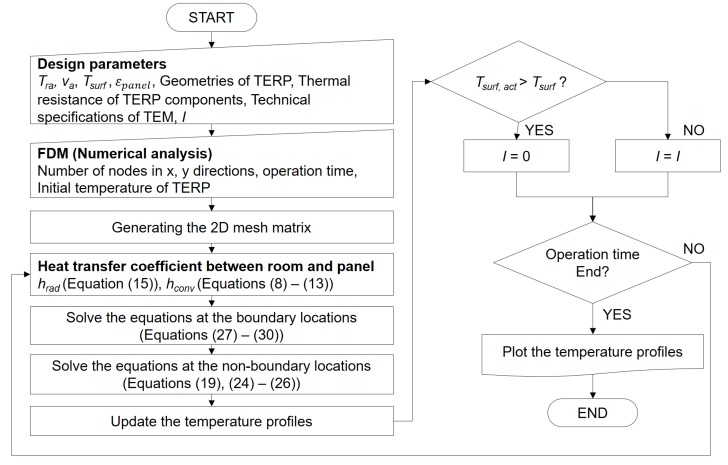
Algorithm of the numerical simulation for the TERP in the heating operation.

For computational calculation, the governing equations were discretized using Equations (24)–(26), and the results are given by Equations (27)–(30), which correspond to Equations (20)–(23). For the stable analysis condition, the time interval (Δ*t*) must be higher than the minimum time step in Equation (31). The minimum time interval purposes to avoid the denominator becoming zero in the equations from the finite difference method (FDM). An algorithm of the whole simulation process is shown in Figure 4. The number of nodes is an input value and it affects the interval of calculation time for numerical simulation.

(24) $$\frac{\partial T}{\partial t}=\frac{T_{i,j}^{t+1}-T_{i,j}^{t}}{\Delta t}.$$

(25) $$\frac{\partial^{2}T}{\partial x^{2}}=\frac{T_{i-1,j}^{t}-2T_{i,j}^{t}+T_{i+1,j}^{t}}{\Delta x^{2}}.$$

(26) $$\frac{\partial^{2}T}{\partial y^{2}}=\frac{T_{i,j-1}^{t}-2T_{i,j}^{t}+T_{i,j+1}^{t}}{\Delta y^{2}}.$$

(27) $$T_{i,j}^{t+1}=T_{i,j}^{t}+\frac{\kappa \Delta t}{\rho C_{p}}\left(\frac{T_{i+1,j}^{t}+T_{i-1,j}^{t}-2T_{i,j}^{t}}{\Delta x^{2}}+\frac{T_{i,j+1}^{t}+T_{i,j-1}^{t}-2T_{i,j}^{t}}{\Delta y^{2}}\right).$$

(28) $$T_{i,j}^{t+1}=T_{i,j}^{t}+\frac{\kappa_{\mathrm{Al}}\Delta t}{\rho_{\mathrm{Al}}C_{p,\;\mathrm{Al}}}\left(\frac{T_{i+1,j}^{t}+T_{i-1,j}^{t}-2T_{i,j}^{t}}{\Delta x^{2}}+\frac{T_{i,j+1}^{t}-T_{i,j}^{t}}{\Delta y^{2}}\right)+\frac{Q_{\mathrm{comb}}\Delta t}{\rho_{\mathrm{Al}}C_{p,\;\mathrm{Al}}\partial x\partial yW_{\mathrm{panel}}}.$$

(29) $$T_{i,j}^{t+1}=T_{i,j}^{t}+\frac{K\Delta t}{\rho_{\mathrm{TEM}}C_{p,\mathrm{TEM}}}\left(\frac{T_{i+1,j}^{t}+T_{i-1,j}^{t}-2T_{i,j}^{t}}{\Delta x^{2}}+\frac{T_{i,j-1}^{t}-T_{i,j}^{t}}{\Delta y^{2}}\right)+\frac{Q_{h,\;\mathrm{TEM}}\Delta t}{\rho_{\mathrm{TEM}}C_{p,\mathrm{TEM}}\partial x\partial yW_{\mathrm{panel}}}.$$

(30) $$T_{i,j}^{t+1}=T_{i,j}^{t}+\frac{\kappa_{\mathrm{Al}}\Delta t}{\rho_{\mathrm{Al}}C_{p,\;\mathrm{Al}}}\left(\frac{T_{i+1,j}^{t}+T_{i-1,j}^{t}-2T_{i,j}^{t}}{\Delta x^{2}}+\frac{T_{i,j+1}^{t}-T_{i,j}^{t}}{\Delta y^{2}}\right)+\frac{Q_{\mathrm{comb}}\Delta t}{\rho_{\mathrm{Al}}C_{p,\;\mathrm{Al}}\partial x\partial yW_{\mathrm{panel}}}.$$

(31) $$Minimum\Delta t=\frac{\rho_{\mathrm{TEM}}C_{p,\mathrm{TEM}}\left(\Delta x^{2}\Delta y^{2}\right)}{2\kappa_{\mathrm{Al}}\left(\Delta x^{2}+\Delta y^{2}\right)}.$$

## 4. Laboratory Experiments

### 4.1. Overview of Experiments

**Figure 5:**
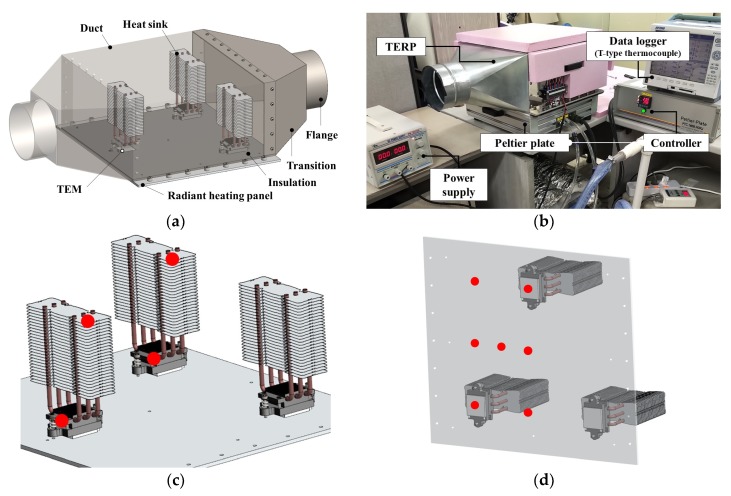
Experimental setup for the TERP: (**a**) three dimensional model of mockup; (**b**) experimental equipment; (**c**) sensors for the heat fin and base plate; and (**d**) sensors for the radiant panel.

A mockup model of the TERP [19] was used for experiments (Figure 5a). Three TEMs were used for TERP whose length and width was 0.4 m with 0.25 m height. The thickness of the radiant heating panel was 5 mm, and Teflon insulation was used [46]. There was a duct to intake air as a heat source, and two transitions were used to connect the flexible duct from the environment chamber to the air side of the TERP. The heat sink comprised of a base plate and a heat fin, and a heat pipe was used to expand the heat-exchange area between the air and the TEM.

The constructed mockup model of the TERP and the experimental equipment are shown in Figure 5b. A switched mode power supply (SMPS) can independently control the direct-current and input voltages. The temperature was measured using T-type thermocouples (Omega Engineering, Inc., Norwalk, CT, USA, Error rate ±0.5 °C) and recorded using a data logger (MV2000, Japan, Yokogawa) whose accuracy is ±0.5 °C in the measuring range from −200 to 400 °C [47]. A total of eleven thermocouples were used, and the measured points are shown in Figure 5c,d. The environment chamber was used to adjust the condition of the air, and a variable-speed fan was used to supply the air to the duct of the TERP. When the air was not necessary, the duct of TERP was closed using a cover.

The Peltier plate was employed to create the room-temperature condition [19]. It works based on the TEMs for achieving the uniform target surface temperature. Four TEMs were arranged with the spacing of 13 and 14 cm in horizontal and vertical directions. The rejected heat from the TEMs was removed using water supplied from the chiller. The input current of TEMs was manipulated based on the proportional-integral differential method to achieve the target value with an accuracy of 0.5 °C.

The heating capacity of the TERP is derived using Equation (32) and is based on the convection and radiation, as indicated by Equations (14) and (16). Therefore, the thermal resistance between the room and the panel surface ($R_{\mathrm{room}-\mathrm{panel}}$) is able to be defined based on the panel area (*A*_panel_) and combined heat transfer coefficient (*h*_comb_) in Equation (33). Additionally, its effect can be replaced with the heat conduction through insulation [19,48]. Consequently, the amount of heat flow from the room air to the panel was controlled according to the property of insulation and surface temperature of the Peltier plate.

(32) $$Q_{\mathrm{panel}}=\left(h_{\mathrm{conv}}+h_{\mathrm{rad}}\right)A_{\mathrm{panel}}\left(T_{\mathrm{panel}}-T_{\mathrm{room}}\right)=h_{\mathrm{comb}}A_{\mathrm{panel}}\left(T_{\mathrm{panel}}-T_{\mathrm{room}}\right).$$

(33) $$R_{\mathrm{room}-\mathrm{panel}}={\left(h_{\mathrm{comb}}A_{\mathrm{panel}}\right)}^{-1}={\left(\kappa_{\mathrm{ins}}\frac{A_{\mathrm{panel}}}{\tau_{\mathrm{ins}}}\right)}^{-1}.$$

### 4.2. Experiment 1: Validation of the Numerical Model

Experiment 1 was performed to validate the developed numerical simulation model for investigating the thermal behavior of the TERP in the heating mode without fan operation. The arrangement of TEM on the surface of mock up TERP may cause a difference between the simulated and measured value to occur, however horizontal heat transfer on the surface of panel has relatively low effects on the thermal behavior of TEM.

**Figure 6:**
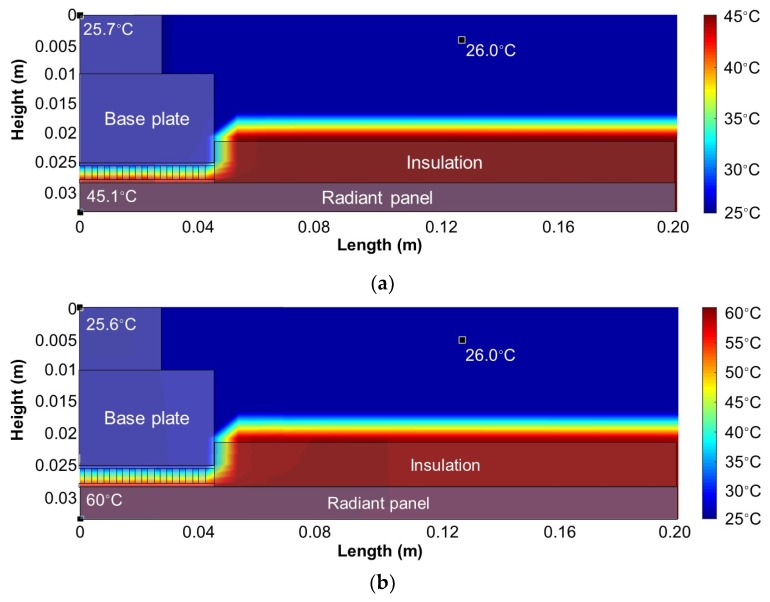
Simulation results for the TERP in heating mode: (**a**) *T*_surf_ = 45 °C and (**b**) *T*_surf_ = 60 °C.

First, two simulations were conducted for the surface temperatures of 45 and 60 °C. The simulation time was 30 min, and the room temperature was set as 24 °C. The air temperature in the duct was set as 26 °C according to the actual temperature of the mockup TERP in the laboratory. The simulation results are shown in cross-sectional 2D color contours for plotting the thermal behavior (Figure 6). The numerical simulation revealed that the surface temperature could be maintained without fan operation (i.e., without the heat source from the cold side of the TEM). Additionally, the cold-side temperature of the TEM was 25.7 and 25.6 °C when the surface temperature was 45 and 60 °C, respectively. These temperatures were similar to that of the air in the duct. Thus, little heat was transferred from the air to the base plate, and the heating was mainly based on the heat generated by the TEM via the Joule effect.

**Figure 7:**
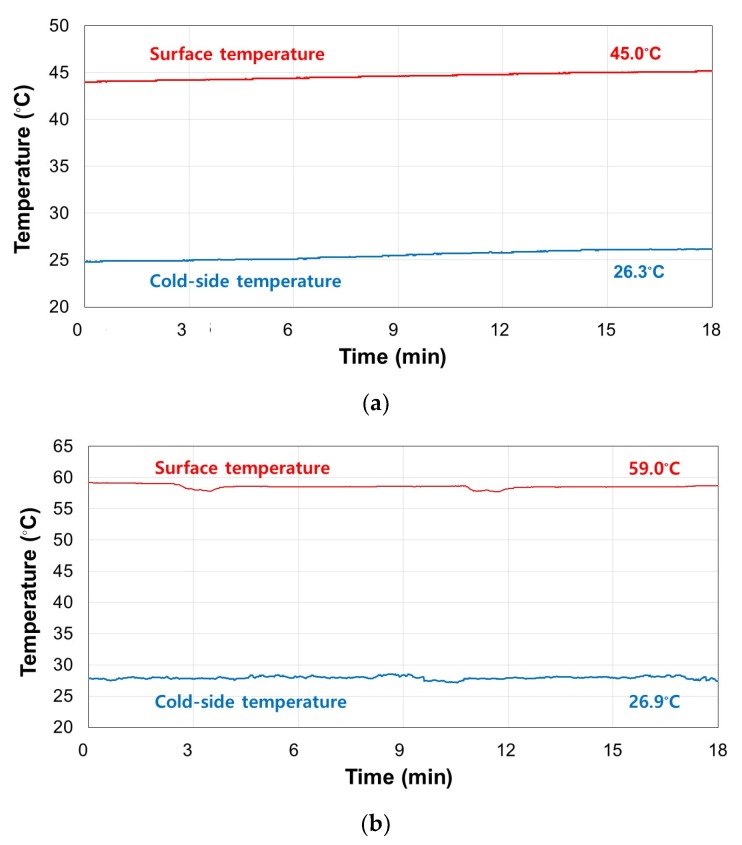
Experimental results for the TERP in heating mode: (**a**) *T*_surf_ = 45 °C and (**b**) *T*_surf_ = 60 °C.

The validation experiments were conducted under the same operation conditions as the numerical simulations. The surface and cold-side temperatures of the TERP were measured, as shown in Figure 7. The target surface temperatures of 45 and 60 °C were maintained stably without fan operation. The cold-side temperatures were slightly higher than the initial air temperature of 26 °C in both cases. However, it is only a 0.6 and 1.3 °C difference with the simulation value. This is because the air in the duct was slightly heated during operation owing to the non-ideal insulation between the aluminum panel and the air in the duct. The purpose of the numerical simulation was to confirm the heating principle of the heating mode without fan operation in the TERP; hence, the error resulting from the incomplete insulation was not much important. Consequently, the thermal behaviors from the numerical simulation and the validation experiment exhibited good agreement. Therefore, we could conclude that the TERP could operate in the heating mode without an air heat source and fan operation via the Joule effect of the TEM.

### 4.3. Experiment 2: Investigation of Energy Consumption by Fan Operation

#### 4.3.1. Experiment Cases

The objective of experiment 2 was to investigate the necessity of the fan operation from the viewpoint of the energy performance of heating operation of TERP. The energy consumption was measured according to three variables: the surface temperature, volume air flow rate, and air temperature in the duct. Using the 2^k^ factorial design method, 10 experiment cases were designed. Eight cases involved a combination of three independent variables, and two cases involved operation without the fan (Table 2). The minimum and maximum air temperatures (*T*_OA_) were 8 and 36 °C, respectively, i.e., the greatest difference possible according to the capacity of the environment chamber. The minimum and maximum surface temperatures (*T*_surf_) were set as 45 and 60 °C, respectively [49,50,51]. The volume air flow rates (${\dot{V}}_{\mathrm{OA}}$) were 50 and 400 m^3^/h, in accordance with a previous study [18]. The flow rate and temperature of air in a duct were adjusted using the environment chamber and a variable-speed fan. The electric power consumptions of the TEMs were easily measured according to the input value of the SMPS, and that of the fan was evaluated using the control panel of the environment chamber. The combined heat transfer coefficient was set to be 6.8 W/m^2^K by using 5-mm-thick expanded polystyrene (EPS) insulation between the plate and the TERP and the room temperature was set as 24 °C using the Peltier plate.

#### 4.3.2. Experimental Results

**Figure 8:**
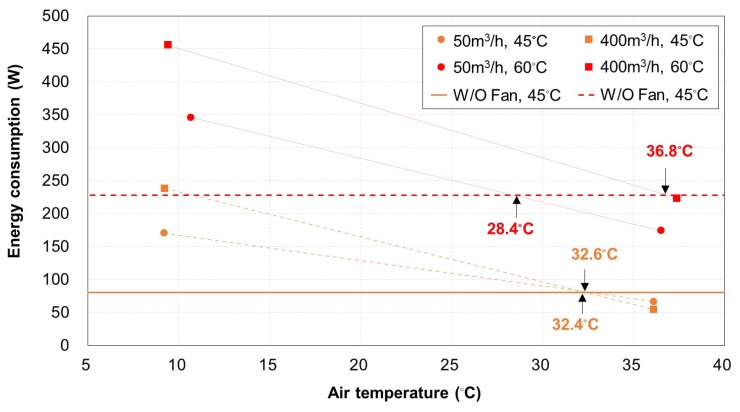
Energy consumption of the TERP in the heating mode according to the volume flow rate and temperature of air.

In Figure 8, the mockup TERP without fan operation consumed 80.1 and 227.9 W for the surface temperatures of 45 °C and 60 °C, respectively. The TERP with fan operation in the heating mode consumed more energy with a higher surface temperature, a lower air temperature, and more volume flow rate of the air in the duct. The higher surface temperature indicated a higher heating capacity; therefore, it is natural that the energy consumption increased. Moreover, the air with low temperature could not supply enough heat to the cold side of TERP, therefore it had better not be used in aspects of energy.

The criteria of *T_OA_* for achieving energy saving by operating the fan were estimated through linear regression between two points. The simple linear regression method could be used based on the linearity between the energy consumption of TEM and ΔT between the hot and cold side of TEM [12,28], since the heating surface temperature of TERP was fixed and the cold side temperature was changed by the air temperature.

It was unnecessary to operate the fan for TERP heating when *T*_OA_ was <32.4 °C regardless with ${\dot{V}}_{OA}$ in a duct. However, TERP operation with ${\dot{V}}_{\mathrm{OA}}$ of 50 and 400 m^3^/h had energy benefits when *T_OA_* was higher than 28.4 and 36.8 °C, respectively. These criteria for *T*_OA_ based on ${\dot{V}}_{\mathrm{OA}}$ were the same when *T*_surf_ was 45 °C. They differed when *T*_surf_ was 60 °C because using increased ${\dot{V}}_{\mathrm{OA}}$ requires more fan energy. When *T*_surf_ was 45 °C, the energy saving for the TEM achieved by increasing ${\dot{V}}_{\mathrm{OA}}$ was almost equal to the additional energy consumption of the fan. Moreover, the energy saving for the TEM achieved by increasing ${\dot{V}}_{\mathrm{OA}}$ was smaller than the additional energy consumption from the fan when *T*_surf_ was 60 °C. As there is no heating season when *T*_OA_ is >28 °C, it can be concluded that fan operation is unnecessary in the heating operation of the TERP.

## 5. Prediction Models for Energy Consumption and Heating Capacity

It was revealed in the previous section that the TEM is only activated for heating in the TERP. As described in this section, empirical models were developed to predict the energy consumption and heating capacity of the TERP in the heating operation. For the experiment design, the 2^k^ factorial design method was employed to develop a regression model using the response surface methodology (RSM) [52].

### 5.1. Model Parameters

Three model parameters were defined as the following independent variables: the target surface temperature (*T*_surf_), room temperature (*T*_RA_), and combined heat-transfer coefficient (*h*_comb_). In Table 3, the valid range of each parameter is presented. The minimum and maximum target surface temperatures (*T*_surf_), room temperatures (*T*_RA_), and the combined heat transfer coefficient (*h*_comb_) had as wide a range as possible, for maximizing the utilization of the empirical models. The minimum and maximum combined heat-transfer coefficients (*h*_comb_) were derived according to the combination of natural and forced convection, for a mean radiant temperature (*T*_MRT_) between 20 and 28 °C.

The target surface temperature was the set value; therefore, the TERP adjusted the surface temperature using the input voltage from the SMPS. If three temperature sensors facing the TEM on the aluminum panel stably indicated the same temperature as the target value, the input voltage could be fixed. The combined heat-transfer coefficient was adjusted using 3- and 5-mm-thick EPS insulation, representing the combined heat-transfer coefficients in forced and natural convection situations, respectively. The room temperature was controlled using the Peltier plate described in Section 4.1. Using all three independent variables, a total of eight experiment cases were derived, as shown in Table 4.

The dependent variables were the required power (*P*) and the heating capacity (*Q*_panel_). The heating capacity of the TERP (*Q*_panel_) was not affected by the properties of TEM; however, the required power (*P*) depended on the TEM. Therefore, the normalized power (P*; defined in Equation (34)) was used for dimensionless modeling. The maximum power (*P*_max_) can be easily calculated using the properties of the TEM. Therefore, the actual required power (*P*) can be derived with the normalized power (P*). This method can be applied when it was postulated that the performance curves of commercial TEMs showed an alike value [19]. The performance curve of the TEM depends on the materials, number of thermocouples, geometry, etc. However, almost all commercial TEMs employ bismuth telluride (Bi_2_Te_3_) for the thermocouples. Additionally, they have similar fractions of the total TEM area covered by thermocouples, despite the different numbers of thermocouples. The heating COP of TEM should be higher than 1.0, which is higher than that of an electric heater owing to the heat from the cold side by the Peltier effect when the normalized power (P*) is higher than 0.05.

(34) $$P^{*}=\frac{P}{P_{\max}}.$$

The heating capacity (*Q*_panel_) of the TERP in the experiments was calculated using Equation (35). The room temperature (*T*_RA_) and combined heat-transfer coefficient (*h*_comb_) were fixed independent variables, and the actual surface temperature (*T*_surf,act_) was the average value for seven thermocouple sensors on the surface of the TERP. It is also specified that the unit of heating capacity of TERP (*Q*_panel_) is watt per square meter (W/m^2^).

(35) $$Q_{\mathrm{panel}}=h_{\mathrm{comb}}\left(T_{\mathrm{surf},\;\mathrm{act}}-T_{\mathrm{RA}}\right).$$

### 5.2. Operation Data

The operation data was collected from the eight experiment cases in Table 4. An experiment case was performed during 10 min and surface temperature was measured with 10 s interval when the TERP showed the stable condition. The purpose of prediction models was estimating the heating capacity and power consumption during the operation of TERP, therefore it was necessary to measure the performance in the stable condition. Consequently, a total of 581 data samples were collected and averaged according to the eight experiment cases. The reason we averaged the data was that the dependent variables of heating capacity (*Q*_panel_) and normalized power (P*) showed mostly a steady value or fixed value, therefore it was not suitable using all raw data for statistical analysis.

The measured heating capacity of TERP (*Q*_panel_) and normalized power (P*) are summarized in Table 5. In the experiment, the system COP of TERP in the heating operation ranged from 0.40 to 1.18 according to the operation condition. The system COP of TERP was lower than that of TEM because of the heat losses to the duct side even though there was insulation. The higher combined heat transfer coefficient (*h*_comb_) showed a higher system COP of TERP, which means the conditions in forced convection. The lower room temperature (*T*_RA_) also presented the higher system COP of TERP because of the higher temperature difference between the room and panel surface.

### 5.3. Uncertainty Analysis

The overall uncertainty was derived to validate the measured values based on the ASHRAE guidelines [53]. There are two errors for the overall uncertainty—the random error (*p*_y_) and the propagation error (*b*_y_)—as described by Equation (36). The random error (*p*_y_) was calculated using Equation (37) with the mean value (*M*) and standard deviation (*S*_r_) of the measured surface temperature. The propagation error (*b*_y_) occurred from a propagation in data reduction. However, there was no data reduction because there was only one parameter (i.e., the surface temperature) to measure. Hence, a fixed error ($b_{x_{i}}$) was the propagation error (*b*_y_) equal to the multiplication of the standard deviation of the measured surface temperature ($S_{r}$) sensor error of the T-type thermocouple in Equation (38). Finally, the overall uncertainty values were varied from 0.023 to 0.180 °C, which are lower than the accuracy of the thermocouple sensor in the eight experiment cases.

(36) $$U_{y}=\sqrt{\left(p_{y}{}^{2}+b_{y}{}^{2}\right)}.$$

(37) $$p_{y}=\frac{2S_{r}}{\sqrt{M}}.$$

(38) $$b_{y}=\sqrt{\left[\sum_{i=1}^{n}{\left(\frac{dy}{dx_{i}}b_{x_{i}}\right)}^{2}\right]}.$$

### 5.4. Model Derivation

Design-Expert ver. 11 [54] was used for the statistical analysis employing the RSM method. Two empirical models were developed as functions of the three design parameters for predicting the heating capacity and energy consumption of TERP. A linear model was applied for both prediction models, and the results are presented in Table 6. In the analysis of variance (ANOVA) table, three parameters were significant for both prediction models, with a *p*-value of <0.05. Additionally, the effect sizes of each design parameter were analyzed. The results indicated that the target surface temperature had the highest effect size for the energy consumption of TERP.

**Figure 9:**
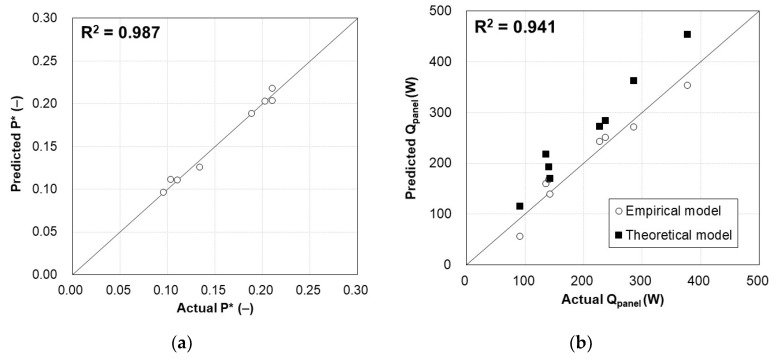
Comparison of the predicted results with experimental results: (**a**) energy consumption of the TERP and (**b**) heating capacity of the TERP.

The prediction models for the heating capacity and energy consumption are given by Equations (39) and (40). The residuals followed a normal distribution well in the normal probability for both developed models without distortion. In Figure 9, the actual and predicted values of the energy consumption and heating capacity were compared. In each model, the predicted results exhibited good agreement with the actual results, with R^2^ values of 0.987 and 0.941 for the energy consumption and heating capacity, respectively. The theoretical heating capacity of the TERP is compared in Figure 9b. The theoretical model was the same as Equation (35), except that the target surface temperature (*T*_surf_) was used instead of the actual surface temperature (*T*_surf,act_). Due to the uneven surface temperature distribution of TERP, the actual surface temperature could not be higher than the target surface temperature in the heating operation. It is natural that the radiant panel had a surface-temperature difference in the heating mode, as ASHRAE recommended that the surface temperature should not exceed 10 °C [55]. This is the necessity of the empirical model, which can predict the heating capacity of TERP, rather than a theoretical model. The developed empirical model was more accurate than the theoretical model, particularly for heating capacities of >300 W.

(39) $$Q_{\mathrm{panel}}=-131.53368+6.90471T_{\mathrm{surf}}+24.55932h_{\mathrm{comb}}-10.36497T_{\mathrm{RA}}.$$

(40) $$P^{*}=-0.15166+0.00615T_{\mathrm{surf}}+0.00333h_{\mathrm{comb}}-0.00185T_{\mathrm{RA}}.$$

### 5.5. Model Validation

Using the center composite design (CCD) method [52], the additional experiment cases for model validation were designed. In the CCD, there are three matrices that consist of the 2^k^ factorial design, the axial point, and the center point. The 2^k^ factorial design matrix was previously used for statistical modeling; therefore, the axial (Case 1–6) and center point (Case 7) matrices were selected for validation experiments, as shown in Table 7.

**Figure 10:**
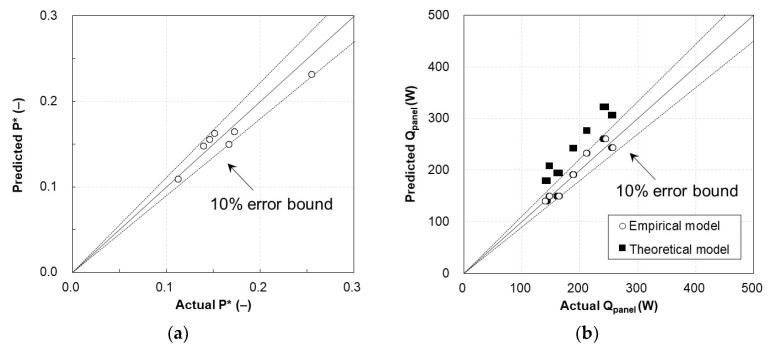
Validation experiment results of the TERP in the heating mode: (**a**) energy consumption of the TERP and (**b**) heating capacity of the TERP.

A total of 2726 data samples were acquired from seven experiment sets for validation. Using the proposed prediction models (Equations (39) and (40)), the predicted energy consumption and heating capacity of the TERP were compared with the measured values, as shown in Figure 10.

The empirical models for predicting the heating capacity and energy consumption of the TERP agreed well (within 10% error bounds) with the values from the measurements. Additionally, the theoretical heating capacity of the TERP is compared in Figure 10b. Similar to the results in Figure 9b, the theoretical model exhibited a larger prediction error.

## 6. Conclusions

In the present study, the suitable operation of the TERP for space heating was investigated using a numerical simulation and experiments. Additionally, empirical models were developed for predicting the energy consumption and heating capacity of the TERP in the heating mode.

The results indicated that TERP showed better performance when it was operated without a fan, which supplies the heat source to the cold side of TEM. It means the low temperature air source in the heating season hinders the heating operation of TERP, and it is better to operate the TERP autonomously. This operation strategy can also avoid the frosting problem without the defrosting process. From the experiment, the system COP of TERP for space heating was ranged from 0.4 to 1.2. The system COP of TERP was similar or slightly higher than that of electric radiator, while it has the advantage that the TERP can be used for heating and cooling both, different with the electric radiator.

The developed prediction models exhibited good agreement (with 10% error bounds) with actual values in validation experiments. The two developed models are dimensionless, and their independent variables can be easily defined; therefore, these prediction models can be used for the design stage and building energy simulation programs. In addition, the prediction model for the energy consumption can be applied on the control logic to determine the input power according to the target surface temperature and room condition. In the future study, the extended study will be conducted to reclaim the waste heat from the exhaust air for heating operation of TERP integrated with a ventilation system.

## Figures and Tables

**Table 1:** Technical specifications of the TEM used in this study.

Description	Value
Dimensions	40 mm × 40 mm × 3.8 mm
Material	Bismuth Telluride (Bi_2_Te_3_)
*I*_max_	6.4 A
*V*_max_	14.7 V
*Q*_max_	56 W
Δ*T*_max_	71 °C

**Table 2:** Experiment cases for examining the necessity of fan operation.

Case	*T*_surf_ (°C)	${\dot{V}}_{\mathrm{OA}}\;(m^{3}/h)$	*T*_OA_ (°C)
1	45	-	-
2	45	50	8
3	45	400	8
4	45	50	36
5	45	400	36
6	60	-	-
7	60	50	8
8	60	400	8
9	60	50	36
10	60	400	36

**Table 3:** Valid ranges of the developed empirical models.

Design Parameter	Low	High
*T*_surf_ (°C)	45	60
*h*_comb_ (W/m^2^K)	6.8	11.3
*T*_RA_ (°C)	20	28

**Table 4:** Experiment cases for developing the empirical model of the TERP in heating mode.

Case	*T*_surf_ (°C)	*T*_RA_ (°C)	*h*_comb_ (W/m^2^K)
1	45	20	6.8
2	60	20	6.8
3	45	28	6.8
4	60	28	6.8
5	45	20	11.3
6	60	20	11.3
7	45	28	11.3
8	60	28	11.3

**Table 5:** Experiment results for developing the empirical model of the TERP in the heating mode.

Case	*Q*_panel_ (W/m^2^)	P* (–)	*COP* (–)
1	118.7 ± 0.65	0.111	0.70
2	189.9 ± 0.23	0.203	0.61
3	76.3 ± 0.30	0.096	0.52
4	112.8 ± 2.46	0.189	0.40
5	240.2 ± 3.91	0.134	1.17
6	377.6 ± 3.31	0.210	1.18
7	141.1 ± 1.69	0.103	0.90
8	285.9 ± 1.54	0.210	0.89

**Table 6:** ANOVA results of the developed empirical models.

**(a) Energy Consumption of the TERP in Heating Mode**						
**Source**	**Sum of Squares**	**df**	**Mean Square**	**F-Value**	***p*-Value**	**Effect Size**
Model	0.0179	3	0.0060	101.04	0.0003	
A: *T*_surf_	0.0170	1	0.0170	288.00	0.0001	0.938
B: *h*_comb_	0.0005	1	0.0005	7.69	0.0492	0.025
C: *T*_RA_	0.0004	1	0.0004	7.44	0.0485	0.024
Residual	0.0002	4	0.0001			
Cor Total	0.0182	7				
**(b) Heating Capacity of the TERP in Heating Mode**						
**Source**	**Sum of Squares**	**df**	**Mean Square**	**F-value**	***p*-value**	**Effect Size**
Model	59994	3	19,998	21	0.0063	
A: *T*_surf_	21,454	1	21,454	23	0.0087	0.337
B: *h*_comb_	24,791	1	24,791	27	0.0067	0.389
C: *T*_RA_	13,749	1	13,749	15	0.0185	0.216
Residual	3734	4	934			
Cor Total	63,728	7				

**Table 7:** Experiments cases for model validation.

Case	*T*_surf_ (°C)	*T*_RA_ (°C)	*h*_comb_ (W/m^2^K)
1	52.5	24	6.8
2	52.5	24	11.3
3	52.5	28	8.5
4	52.5	20	8.5
5	60	24	8.5
6	45	24	8.5
7	52.5	24	8.5
